# Noble Metal Nanoparticles for Point-of-Care Testing: Recent Advancements and Social Impacts

**DOI:** 10.3390/bioengineering9110666

**Published:** 2022-11-08

**Authors:** Keven Luciano, Xiaochuan Wang, Yaning Liu, Gabriella Eyler, Zhenpeng Qin, Xiaohu Xia

**Affiliations:** 1 Department of Chemistry, University of Central Florida, Orlando, FL 32816, USA; 2 School of Social Work, College of Health Professions and Sciences, University of Central Florida, Orlando, FL 32816, USA; 3 Department of Mechanical Engineering, University of Texas at Dallas, Richardson, TX 75080, USA; 4 Department of Bioengineering, Center for Advanced Pain Studies, University of Texas at Dallas, Richardson, TX 75080, USA; 5 Department of Surgery, University of Texas Southwestern Medical Center, Dallas, TX 75390, USA

**Keywords:** point-of-care test, disease biomarker, noble metal, nanoparticle, social impact

## Abstract

Point-of-care (POC) tests for the diagnosis of diseases are critical to the improvement of the standard of living, especially for resource-limited areas or countries. In recent years, nanobiosensors based on noble metal nanoparticles (NM NPs) have emerged as a class of effective and versatile POC testing technology. The unique features of NM NPs ensure great performance of associated POC nanobiosensors. In particular, NM NPs offer various signal transduction principles, such as plasmonics, catalysis, photothermal effect, and so on. Significantly, the detectable signal from NM NPs can be tuned and optimized by controlling the physicochemical parameters (e.g., size, shape, and elemental composition) of NPs. In this article, we introduce the inherent merits of NM NPs that make them attractive for POC testing, discuss recent advancement of NM NPs-based POC tests, highlight their social impacts, and provide perspectives on challenges and opportunities in the field. We hope the review and insights provided in this article can inspire new fundamental and applied research in this emerging field.

## 1. Introduction

Point-of-care (POC) testing can be informally defined as a rapid way to make a medical diagnosis close to the point at which the test is taken [1,2,3]. One of the first documented POC tests was developed in the 1960s to quantify blood glucose levels [4,5,6]. Through time, POC tests became more and more common, such as the at-home pregnancy test, which was first introduced in the 1970s and commercialized in the 1980s [7,8]. Modern-day examples of POC tests include lateral flow assays (LFAs), electrochemical biosensors, dipsticks, and many others [9,10,11]. To standardize POC tests, the World Health Organization designed the ASSURED criterium to judge a test’s affordability, sensitivity, specificity, user-friendliness, rapidness, equipment, and deliverability.

The importance of POC testing lies in its ability to provide the early detection of infectious and noninfectious diseases alike. The ongoing coronavirus disease 2019 (COVID-19) pandemic highlights the critical need for POC testing [12,13,14,15]. Many laboratory tests, such as polymerase chain reaction (PCR) and mass spectroscopy, are tedious, expensive, and require trained professionals to operate the tests. In contrast, POC tests offer a simple, tactile, and straightforward method, to deliver medical prognosis to patients quickly and effectively. The benefits of POC testing over laboratory tests is prevalent around the world and provides a promising future for the early, sensitive diagnosis of a wide array of illnesses. For instance, converting from lab tests to POC tests for large-scale screening could avert millions of deaths every year in low- to middle-income countries [16,17]. In the United Kingdom, for example, cardiovascular disease testing costs were reduced from EUR 25 to EUR 18 GBP when POC tests were prioritized [16]. The COVID-19 antigen home test (which is based on the LFA platform) can return the results in just 15–30 min.

**Figure 1:**
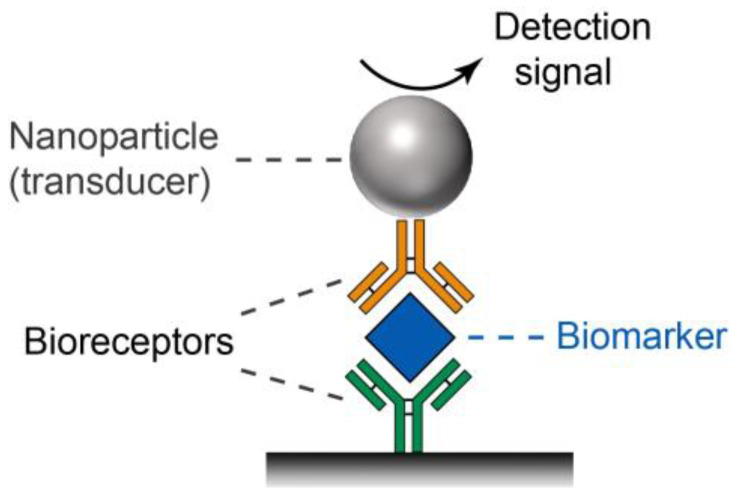
Schematics showing the principle of a typical POC nanobiosensor.

With the rapid advancement of nanoscience and nanotechnology, nanobiosensors have emerged as a robust and effective diagnostic technique in the past couple of decades [18,19,20,21,22,23]. Many nanobiosensors are designed to be simple, rapid, and low-cost, making them particularly suitable for POC testing [24,25]. In a typical setup of a POC nanobiosensor (see Figure 1), bioreceptors (e.g., antibodies and DNAs)-functionalized nanoparticles specifically capture disease biomarkers and generate detectable signal through various transduction mechanisms. As such, the concentration of disease biomarkers in a sample can be quantitatively or qualitatively analyzed by measuring the intensity of detection signal. It should be emphasized that the nanoparticle as signal transducer is a key component of a nanobiosensor, because it is responsible for signal generation and thus largely determines the performance (e.g., sensitivity and reproducibility) of the associated nanobiosensor.

Among various nanoparticles used for POC nanobiosensors, the nanoparticles of noble metals (including gold (Au), silver (Ag), platinum (Pt), palladium (Pd), rhodium (Rh), iridium (Ir), and ruthenium (Ru)) have drawn increasing attention [26,27,28]. The intriguing and superior physicochemical properties of noble metal nanoparticles (NM NPs) make them suitable signal transducers for POC nanobiosensors. For instance, NM NPs provide multiple signal transduction principles (e.g., plasmonic, catalysis, photothermal effect etc.). Significantly, the signal from NM NPs is strong and reliable. More details about the merits and unique features of NM NPs are discussed in Section 2 below. It should be noted that, although the unit prices of noble metals are relatively high, the material cost of NM NPs in the application of POC tests should not be a major concern, because of the tiny amount of usage (typically 10^−6^–10^−9^ g NM NPs per test).

In this article, we discuss recent advancements of NM NPs-based nanobiosensors for POC testing and highlight their social impacts. This article is not meant to cover the full landscape of NM NPs-based POC testing, but primarily focus on recently innovative designs, where most examples highlighted were reported in the past 5 years. We start with the introduction of the unique features of NM NPs that make them appealing for POC testing. Then, we discuss the recent progress in the development of NM NPs-based POC testing. We also elaborate the social impact of NM NPs-based POC testing on addressing critical social issues, such as healthcare disparities, and the management of health care at the individual and community levels. At the end of this paper, we provide our perspectives on the challenges and opportunities in this niche field.

## 2. The Unique Features of Noble Metal Nanoparticles (NM NPs)

Noble metal nanoparticles (NM NPs) have many unique features that make them attractive for the development of advanced nanobiosensors for POC testing.

**(*i*) Intriguing Properties.** NM NPs offer multiple signal transduction principles for POC testing. They can produce various types of detection signal, including: plasmonic, optical, photothermal, colorimetric, electrochemical, surface-enhanced Raman scattering (SERS), and fluorescent signals [26]. Significantly, the signals from NM NPs often outperform those from conventional materials, which allows for highly sensitive detection. For instance, when used as labels, Au NPs of 40 nm in diameter offer much stronger colorimetric signal than dyes, because their absorption cross-section is five orders larger than ordinary organic dyes [29]. The ability of Ag NPs in enhancing Raman signal is orders of magnitude stronger than most non-noble metal NPs [30,31].

**Figure 2:**
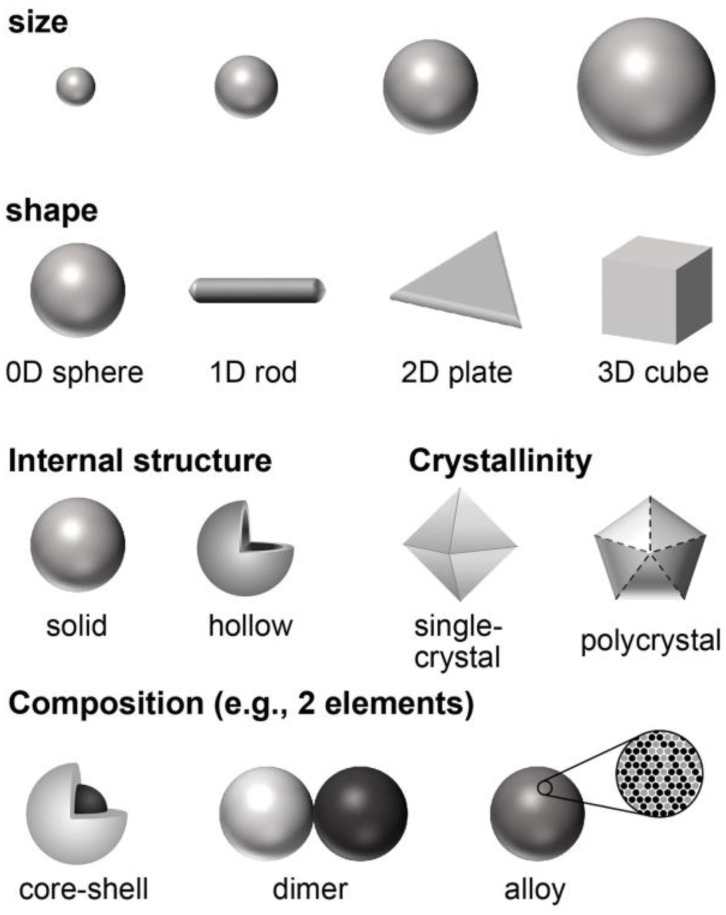
Schematics showing the physicochemical parameters of NM NPs that can be controlled during a synthesis.

**(*ii*) Tunable Physicochemical Parameters.** The properties of NM NPs can be tailored and optimized by controlling their physicochemical parameters (Figure 2) such as size, shape, internal structure (e.g., solid versus hollow), crystallinity (e.g., single crystal versus polycrystal), and elemental composition [32,33]. Taking plasmonic property as an example, 50 nm Au nanospheres display a major localized surface plasmon resonance (LSPR) peak at ~525 nm, while the major LSPR peak of 50 nm × 10 nm Au nanorods is located at ~825 nm [34,35]. The plasmonic activity of Pd NPs in wavelengths of visible light can be substantially enhanced when they are re-shaped from spheres to thin plates [36]. With increased mechanistic understanding on the behaviors of nanocrystal growth and the aid from modern characterization tools (e.g., high-performance electron microscopes), most of these physicochemical parameters can now be precisely controlled in experiments.

**(*iii*) Facile Synthesis.** Thanks to the contributions from multiple research groups in the last several decades, a variety of methodologies have been established for the synthesis of NM NPs [37,38,39]. Particularly, solution-phase synthesis is considered a simple and effective approach for the production of NM NPs with good dispersibility in water [32], which is desired for biomedical applications. Solution-phase synthesis can be performed in an ordinary wet chemistry laboratory without the need of sophisticated instruments. In a typical synthesis, metal precursor is reduced by a reductant in solution in the presence of a colloidal stabilizer. By manipulating thermodynamic and kinetic conditions of a solution-phase synthesis, the growth pathway of nanocrystals and thus the parameters of final products can be controlled. More details about solution-phase synthesis of NM NPs can be found in our recently published review articles [40,41].

**(*iv*) Convenient Surface Functionalization.** The surface of NM NPs can be conveniently functionalized with biomolecules (e.g., proteins, peptides, and nucleic acids), facilitating the application in POC testing. The functionalization can be readily achieved through non-covalent or covalent methods. In non-covalent methods, biomolecules are absorbed to NM NP surfaces through attractive electrostatic interactions at specific pH values [42]. The covalent conjugation of biomolecules to NM NPs can be conveniently achieved by means of metal-thiolate bonding, where a thiol-containing molecule (e.g., thiol-PEGs) is used as a linker to bridge NPs and biomolecules [43,44,45].

**(*v*) Excellent Stabilities.** NM NPs display excellent stabilities because they are made of noble metals that are chemically and thermally inert. For instance, NM NPs have outstanding resistance to oxidation [26,46]. NM NPs have higher melting points compared to most other nanomaterials. For example, Pd nanocubes of 18 nm in edge length could maintain a cubic shape after annealing at 400 °C for 8 min [47]. The thermal stabilities of NM NPs can be further improved by controlling their morphologies and/or compositions. The superior stabilities of NM NPs ensure good consistency of signal production and thus reliable performance of associated POC nanobiosensors.

## 3. Recent Advancements in NM NPs-Based POC Testing

NM NPs have been used for POC testing for decades. The most known example might be the lateral flow assay (LFA, or test strip), where Au NPs are usually utilized as colorimetric labels owing to their outstanding optical properties [48,49]. Over-the-counter pregnancy tests and the recent COVID-19 antigen rapid tests are representative examples of the LFA. Over the last couple of decades, engineered NM NPs have been extensively used for the POC tests of various platforms beyond the LFA, despite most of them being in early stages of commercialization. This section highlights recent NM NPs-based POC testing techniques with innovative designs.

### 3.1. Catalytically Active NM NPs-Based POC Tests

Among NM NPs, platinum-group metal (including Pt, Pd, Rh, Ir, and Ru) NPs are known to be excellent catalysts for many industrially important reactions. In recent years, these catalytic NM NPs have been employed to catalyze reactions that produce detectable signal for POC testing.

**Figure 3:**
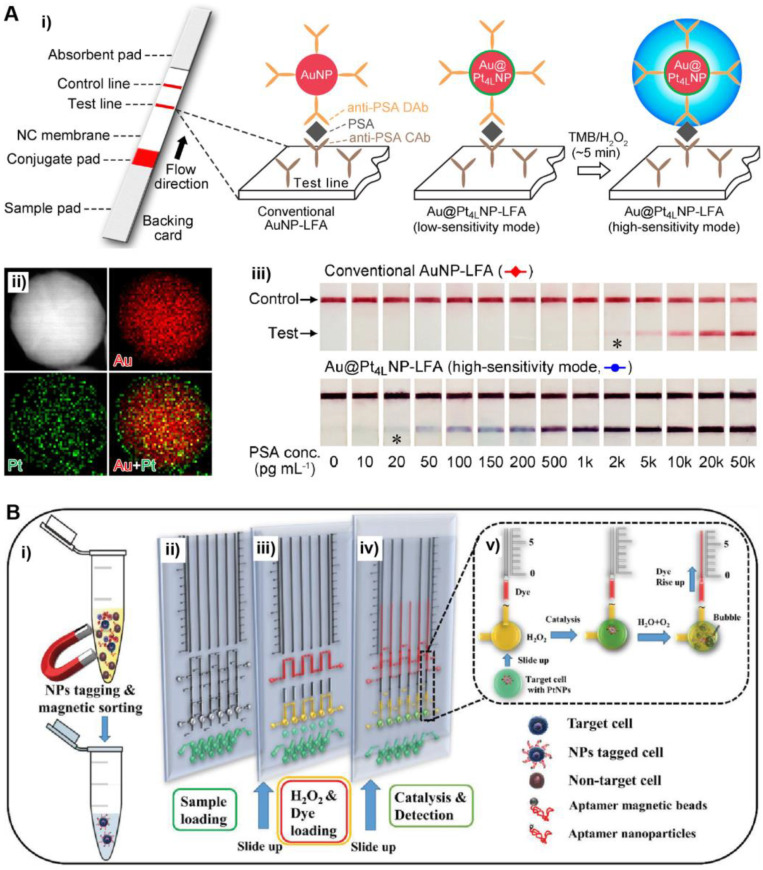
Catalytically active NM NPs-based POC tests. (**A**) Au@Pt core@shell NPs-based LFA: (**i**) schematics showing the detection principles of conventional Au NP- and Au@Pt NP-based LFAs; (**ii**) energy-dispersive X-ray (EDX) mapping image of an individual Au@Pt NP; (**iii**) detection results of the Au NP- and Au@Pt NP-based LFAs of PSA standards. The asterisks (*) indicate detection limits by the naked eyes. Adapted with permission from ref [50]. Copyright 2017 American Chemical Society. (**B**) Pt NPs with volumetric bar chart chip for detection of CTCs: (**i**) sample preparation and aptamer conjugation; (**ii**) sample loading (green); (**iii**) loading of H_2_O_2_ (yellow) and ink (red); (**iv**) Pt NPs-catalyzed decomposition of H_2_O_2_; (**v**) formation of oxygen bubble that displaces the red ink into the vertical parallel channel. Adapted with permission from ref [54]. Copyright 2019 Wiley-VCH.

In a recent work by Xia et al. (Figure 3A), conventional Au NPs of ~40 nm in diameter were coated with a thin layer of Pt to form Au@Pt core@shell NPs [50]. The Au@Pt NPs were able to effectively catalyze the oxidation of 3,3′,5,5′-tetramethylbenzidine (TMB, a typical peroxidase substrate) by H_2_O_2_, producing a blue-colored product oxidized TMB with a large molar extinction coefficient of 3.9 × 10^4^ M^−1^ cm^−1^ [51,52]. The catalytic reaction can be conveniently performed in aqueous solution at room temperature, making it suitable for POC testing. Significantly, the color signal from Au@Pt NPs-catalyzed reaction is much stronger than the color signal from plasmonics of Au NPs, allowing for highly sensitive colorimetric detection. The Au@Pt NPs as labels were applied to the LFA platform. Using human prostate-specific antigen (PSA, a biomarker of prostate cancer) as a model disease biomarker, the Au@Pt NPs-based LFA achieved a low “naked eye” detection limit of 20 pg/mL, which was two orders of magnitude lower than that of conventional Au NPs-based LFA. In another work, Stevens et al. utilized porous Pt NPs to catalyze the oxidation of CN/DAB (4-chloro-1-naphthol/3,3′-diaminobenzidine, tetrahydrochloride) by H_2_O_2_ that generates black-colored products. The Pt NPs were applied to the LFA of p24 (a biomarker of HIV), achieving a low detection limit at the low femtomolar range [53]. Notably, this LFA system was successfully applied to the analyses of clinical human plasma samples.

NM NPs can also be utilized to catalyze reactions that generate signals other than color. Yang et al. reported an innovative POC testing system for circulating tumor cell (CTC) detection that was designed based on the oxygen gas generated by Pt NPs [54]. Specifically, in this system (Figure 3B), target CTCs were captured and labeled with aptamer-conjugated Pt NPs. The Pt NPs can effectively catalyze the decomposition of H_2_O_2_, producing oxygen gas (O_2_). A portable volumetric bar chart chip (V-Chip) was coupled to the detection system. In the presence of target CTCs, the produced O_2_(g) results in movement of an ink bar in the V-Chip. As a result, the number of CTCs in a sample could be conveniently quantified by recording the distance moved by the ink. Such a portable POCT system was sensitive enough for single cell detection. In another design, O_2_(g) generated by NM NPs (e.g., Pt NPs and Au@AgPt NPs) was retained in a confined space [55]. An increased amount of O_2_(g) led to an increase in gas pressure that could be read by a portable pressuremeter. As such, the concentration of target analytes could be quantitively determined by measuring the gas pressure.

### 3.2. Plasmonically Active NM NPs-Based POC Tests

**Figure 4:**
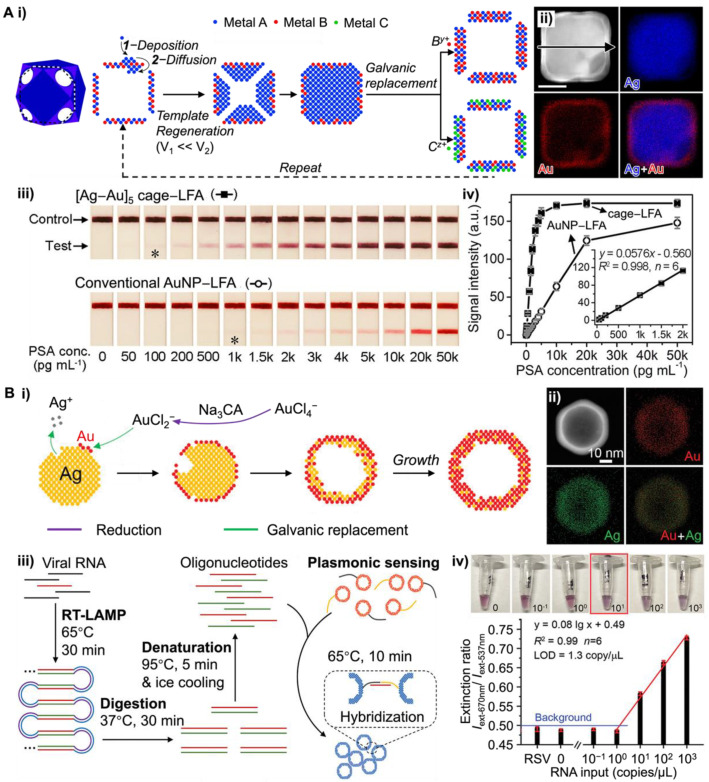
NM NPs with improved plasmonic resonance enabled ultrasensitive POC testing. (**A**) Au-Ag nanocages-based LFA: (**i**) schematic illustration showing the approach of template regeneration and galvanic replacement for the synthesis of metallic nanocages with controlled wall thicknesses; (**ii**) EDX mapping images of an individual Ag@Au-Ag core@shell nanostructure; (**iii**) LFAs for PSA detection using advanced Au-Ag nanocages and conventional Au NPs, respectively. The asterisks (*) indicate detection limits by the naked eyes; (**iv**) calibration curves of the detection results in (**iii**) by quantifying the intensity of testing lines against PSA concentrations. Adapted with permission from ref [60]. Copyright 2020 American Chemical Society. (**B**) Au-Ag nanoshells-based plasmonic LAMP: (**i**) schematics showing the simplified growth of the hollow nanoshells by galvanic replacement; (**ii**) EDX mapping images of an individual Au-Ag shell; (**iii**) concept of the plasmonic LAMP for viral RNA detection; (**iv**) plasmonic LAMP achieved a limit of detection at 1.3 copy/μL for SARS-CoV-2 RNA. Adapted with permission from ref [63]. Copyright 2022 Wiley-VCH.

Plasmonic NM NPs (e.g., Au and Ag NPs) have found wide applications in POC tests [56]. Bimetallic nanostructures, such as gold-silver nanocages, have attracted significant research interest due to the tunable LSPR properties [57,58]. Particularly, their refractive index sensitivity can be effectively regulated by the wall thickness and ratio of Au to Ag. Conventional Au-Ag cages prepared by the galvanic replacement between Ag NPs as templates and HAuCl_4_ are confined to a specific wall thickness [59]. Gao et al. adopted a template regeneration strategy in galvanic replacement reaction to craft the Au-Ag nanocages with controllable wall thicknesses and intriguing plasmonic properties (see Figure 4Ai) [60]. Particularly, the wall of nanocages can be controlled to the desired thickness using regenerated templates (i.e., Ag@Au-Ag core@shell nanostructures, Figure 4Aii) for continuous galvanic replacement. With the well-defined multiwall morphologies and the disappearance of the surface cavities, the LSPR of newly developed Au-Ag nanocages shifted from 775 nm to the visible range of 551 nm. To demonstrate the potential application in POC testing, [Ag-Au]_5_ nanocages (i.e., nanocages of five-layered walls) with λ_max_ of ~550 nm (red color) were applied as labels to the LFA to detect the human prostate-specific antigen (PSA). The results suggested that [Ag-Au]_5_ nanocages achieved a naked eye detection limit at 0.1 ng mL^−1^, which was ~10 times lower than that of conventional Au NP-based LFA (Figure 4Aiii–iv).

Plasmonic coupling assays (PCAs) are another class of rapid tests for a broad range of analytes from proteins to virus particles. The LSPR of NM NPs shifts when NPs come in close proximity to each other (e.g., aggregations) and gives an observable color change. Since the initial report by Mirkin et al. in 1997 [61], NM NPs-based PCAs have been extensively employed in various sensing applications, including the sample-to-answer detection of aptamers, proteins, viruses, and bacteria, in diverse biologically complex media to diagnose infectious diseases [62]. Previous work has demonstrated that the plasmonic properties of MN NPs have strong dependence on various parameters, such as their size, morphologies, the composition of metal, and the surrounding environments. Recently, Ye et al. developed a simpler method for preparing Au-Ag nanoshells with enhanced plasmonic activities [63]. Rather than repeating the galvanic replacement reaction on the regenerated templates, they performed the reaction in the presence of Na_3_CA. Upon injecting the HAuCl_4_, the Na_3_CA quickly reduced the Au^3+^ ions into Au^+^, such that the stoichiometry between Au and Ag in the galvanic replacement reaction changed from 1:3 to 1:1 (Figure 4Bi). The resulting Au-Ag nanoshells with hollow interiors show superior plasmonic activities due to the field enhancement from the plasmon hybridization between the inner and outer surfaces. The Energy-dispersive X-ray (EDX) mapping image of an individual Au-Ag nanoshell confirmed the elemental distribution, where Au and Ag elements are diffused throughout the NPs (Figure 4Bii). Compared with the same size solid Au NPs (50 nm) at the same particle concentration, Au-Ag nanoshells have four times higher extinction cross-section at visible wavelength range and 20-fold improvement in detecting DNA. When integrating with reverse transcription loop-mediated isothermal amplification (RT-LAMP, Figure 4Biii), Au-Ag nanoshells realized the single-molecule detection of severe acute respiratory syndrome-related coronavirus 2 (SARS-CoV-2) RNA with high specificity (Figure 4Biv). Liu et al. further demonstrated that altering nanoparticle morphology has a significant importance on the intact virion detection [64]. With respiratory syncytial virus, they demonstrated that Au nanourchins have increased capability to bind to the virus particle compared with spherical Au NP, and stronger plasmonic coupling at longer distances (~10 nm) that are relevant for immunorecognition.

### 3.3. Photothermally Active NM NPs-Based POC Tests

**Figure 5:**
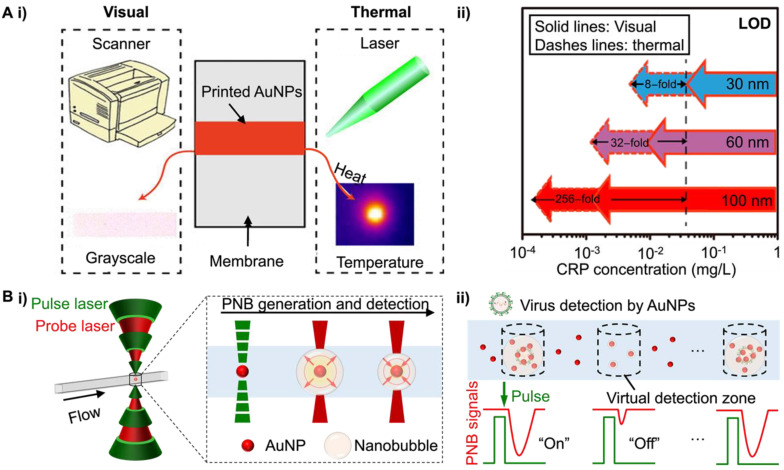

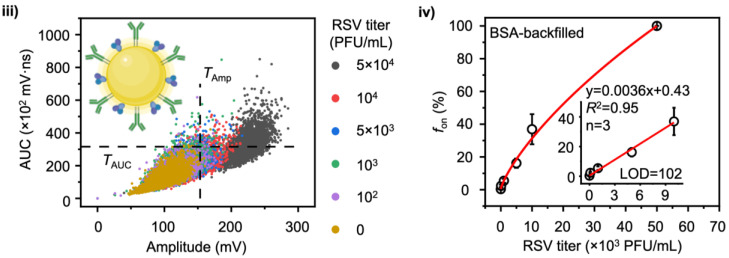
Photothermally active NM NPs for POC tests. (**A**) Thermal contrast amplification for Au NPs-based LFA: (**i**) schematic showing the visual and thermal detection of printed Au NPs on LFA membrane; (**ii**) comparison of the visual and thermal detection sensitivities in the diagnosis of C-reactive protein (CRP) using printed Au NPs of different sizes (e.g., 30, 60, and 100 nm). Adapted with permission from ref [66]. Copyright 2017 American Chemical Society. (**B**) Digital plasmonic nanobubble (PNB) detection for POC diagnosis of RSV: (**i**) schematic illustration of PNB generation mechanism; (**ii**) compartment-free digital plasmonic counting principle for virus detection; (**iii**) bivariate scatter plots of amplitude and area under the curves (AUC) extracted from 3000 PNB signals for RSV detection; inset shows the model of antibody-functionalized Au NPs for the assay; (**iv**) the “f_on_” counting results from (**iii**) against RSV with different concentrations. Adapted with permission from ref [67]. Copyright 2022 Springer Nature.

The absorption of light energy by NM NPs leads to photothermal heating and can serve as sensitive contrast. Qin et al. first reported a thermal contrast amplification (TCA) strategy for Au NP-based LFAs with continuous wave laser heating [65]. By applying laser on a completed LFA strip, the accumulated Au NPs on the test line induce temperature changes that can be directly recorded by an infrared camera or sensor. Compared with visual detection, TCA readout provides improved ability in the analytical quantification of LFA results (Figure 5Ai) [66]. Later optimization of the immunoassays and miniaturization of the TCA instrumentations by Zhan et al. further enhanced the LFA sensitivity up to 256-fold (Figure 5Aii) [66]. Notably, the design of NM NPs as thermal contrast labels has a significant impact on the LFA reaction kinetics and TCA signal, thus affecting the LFA analytical performance. For example, the larger Au NPs hold higher binding affinity to the target analyte due to more antibody conjugation on the Au NP and increased Au NP capture. Combined with the high light absorption and scattering for larger Au NP, they allow much more sensitive detection. Other factors, such as the low diffusion limit for large NPs and highly non-specific background signals caused by membrane-trapping, should also be considered.

While the continuous wave (CW) laser heating leads to a bulk temperature increase, pulsed laser can excite the NM NPs locally and vaporize water to create nanobubbles, referred to as plasmonic nanobubbles (PNBs). Liu et al. utilized the digital PNB (dPNB) detection for intact virus diagnosis [67]. Since the vapor and liquid water have very different refractive indexes, dPNB can be easily detected by a continuous laser probe (Figure 5Bi). An optofluidic setup was designed to flow the Au NP suspensions in a micro-capillary for high throughput detection. The focused laser beams create a microscale “virtual detection zone” of about 16 pL and detect dPNB signals (Figure 5Bii). There is no crosstalk between laser pulses since PNB only last hundreds of nanoseconds. This allows for the rapid counting of dPNBs and set thresholds for “on” and “off” signals in a compartment-free manner. When implemented in a homogeneous assay for respiratory syncytial viruses (RSV) detection, dPNB achieved a limit of detection at ~100 PFU/mL or 1 genome-equivalent copy/µL (Figure 5Biii, iv). This is competitive with nucleic acid amplification methods. Further advantages include the simplicity of the assay without separation or amplification steps, room temperature operation, and rapid dPNB counting, within minutes. Such a system opens new possibilities to develop separation-, amplification-, and compartment-free NM NP-based digital assay that is a rapid and ultrasensitive POC diagnostic platform.

### 3.4. SERS Active NM NPs-Based POC Tests

The Raman signal of molecules can be drastically enhanced by metallic nanoparticles (particularly Ag and Au NPs) owing to the localized electromagnetic field around the surface of NPs [68]. This phenomenon is known as surface-enhanced Raman scattering (SERS), whereas the NPs are called SERS substrates [69]. Since the pioneer work by Van Duyne et al. in 1977, SERS has been broadly used for biosensing applications [70,71]. The recent development of portable or handheld Raman spectrometer makes SERS suitable for POC testing.

In the 2000s and early 2010s, great effort in the field of SERS biosensors had been put on engineering sensitive SERS substrates with large enhancement factors (EFs). In particular, EF of a substrate can be substantially increased through the formation of hot spots (i.e., small, localized regions with intensified electric fields [72]). Common methods for the fabrication of hot spots include engineering nanostructures with sharp features (e.g., corners and edges) and inducing nanoparticle aggregations [73].

**Figure 6:**
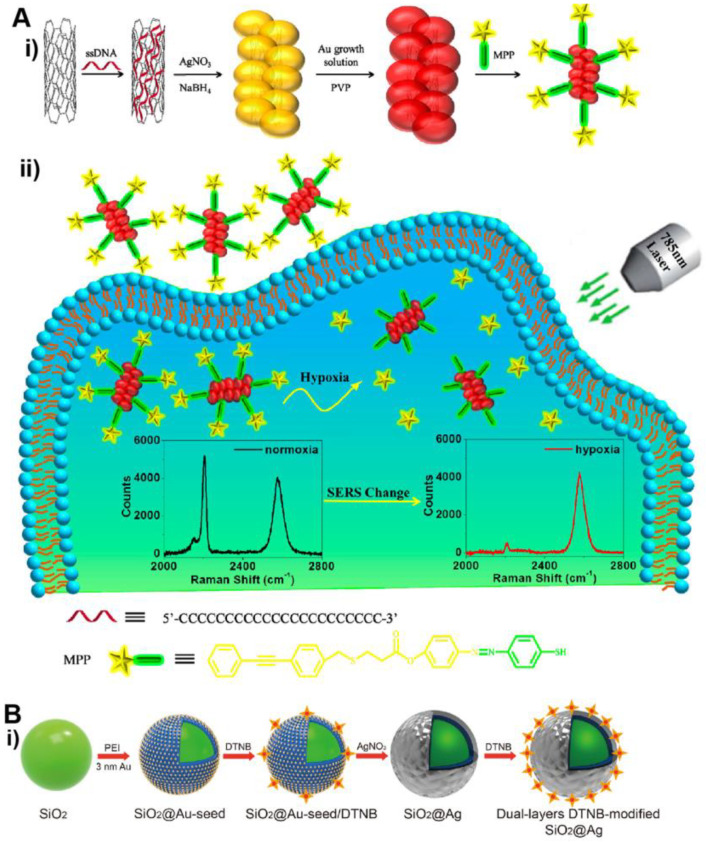

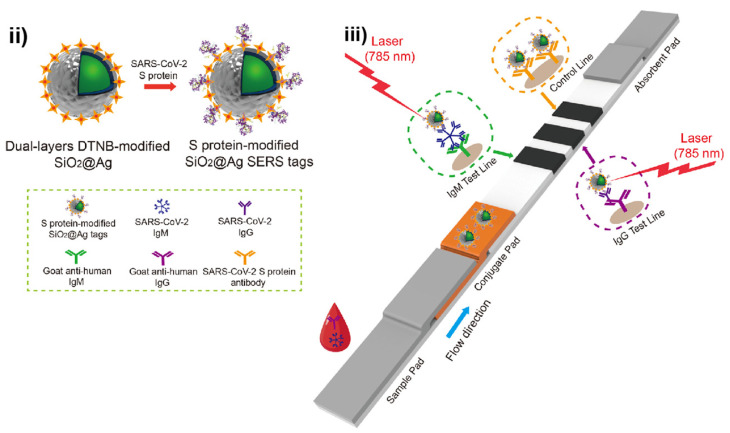
SERS active NM NPs-based POC tests. (**A**) SWCNT/Ag/AuNPs conjugates for SERS imaging of hypoxia: (**i**) preparation of the SWCNT/Ag/AuNPs conjugate-based SERS nanoprobe; (**ii**) sensing principle of hypoxia. Adapted with permission from ref [76]. Copyright 2019 American Chemical Society. (**B**) SERS-based LFA for detection of anti-SARS-CoV-2 IgM and IgG: (**i**) preparation of the dual-layers DTNB-modified SiO_2_@Ag NPs. DTNB = 5,5’-dithiobis-(2-nitrobenzoic acid); (**ii**) SARS-CoV-2 S protein-modified SiO_2_@Ag SERS tags; (**iii**) detection principle of the SERS-based LFA of anti-SARS-CoV-2 IgM and IgG. Adapted with permission from ref [77]. Copyright 2021 Elsevier B.V.

In recent years, the trend of fabricating hybrid SERS substrate has drawn increasing attention, where NM NPs are incorporated with secondary functional materials [74]. Hybrid SERS substrates can integrate the merits of multiple materials and/or produce synergies. For instance, by coupling NM NPs with semiconductors, SERS EF can be enhanced by ~10–10^3^-fold through combined (synergistic) contributions from both materials. In a typical hybrid noble metal-semiconductor system, photoexcited electrons arising from the LSPR of metal flow to conduction band of semiconductor. Such a process promotes a semiconductor-to-molecule charge transfer process, resulting in a chemical mechanism-based SERS enhancement [74]. This synergistic enhancement had been demonstrated in the Au-TiO_2_ system [75]. In another example (Figure 6A), noble metal was coupled with carbon nanotubes [76]. Specifically, single-walled carbon nanotubes (SWCNTs) were functionalized with Ag/Au alloyed NPs to form SWCNT/Ag/AuNPs conjugates. The 2D-band of SWCNTs at 2578 cm^–1^ remains unchanged and thus can be used as the internal reference. This hybrid SERS substrate allows for more reliable and reproducible detection because the signal is measured by ratiometric intensity between SWCNT as an internal reference and a Raman reporter molecule (e.g., MPP with a peak at 2207 cm^−1^).

Another important progress of SERS active NM NPs-based POC testing is to address emerging healthcare issues. A notable strategy is to use SERS tags (i.e., SERS active NPs pre-functionalized with reporter molecules with known Raman peaks) as labels for the LFA. As a distinct advantage over conventional LFAs, SERS tag-based LFA is more sensitive because a small amount of SERS tags specifically captured in the test line of LFA strip can provide strong Raman signal. In a recent study by Wang et al. (see Figure 6B), Raman dye-functionalized SiO_2_@Ag core@shell NPs were used as SERS tags for LFA of anti-SARS-CoV-2 (the virus that causes COVID-19) IgM and IgG [77]. The SERS signal intensities of the IgM and IgG test lines were conveniently recorded by a portable Raman instrument. The detection limit of this SERS tag-based LFA was 800 times lower than that of standard Au NPs-based LFA. Significantly, the SERS tag-based LFA was successfully applied to serum samples collected from COVID-19 patients, demonstrating the potential clinical use of the new technology. The platform of SERS tag-based LFA can also be applied to detection of other infectious diseases. For instance, Choo et al. developed a SERS LFA for serodiagnosis of scrub typhus, a mite-borne infectious disease [78].

### 3.5. Label-Free Colorimetric NM NPs-Based POC Tests

Owing to the outstanding optical properties, NM NPs (especially Au and Ag) have been demonstrated to be excellent colorimetric labels for POC testing where the detection results can be visualized by naked eyes. Importantly, the color of Au and Ag NPs can be tuned in the visible light spectrum by controlling NP morphology (e.g., size and shape) and/or elemental composition [79,80], which allows for the design of innovative POC tests, such as those capable of multiplexed detection.

In recent years, label-free colorimetric NM NPs have been utilized for the development of versatile and sensitive POC tests [81]. In this system, colorimetric NM NPs are not labeled with bioreceptors, which reduces the non-specific binding of NPs caused by bioreceptors and improves detection reproducibility. In a typical design, target analytes in an assay are linked to the generation of certain substance that can trigger the morphological or compositional changes of colorimetric NPs through creative mechanisms (e.g., growth and etching of NPs).

**Figure 7:**
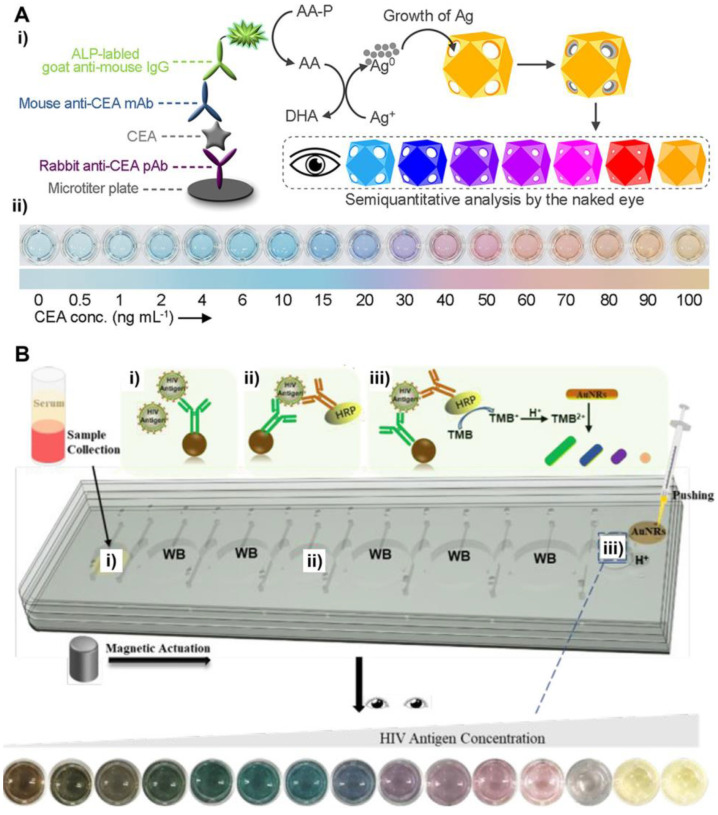
Label-free colorimetric NM NPs-based POC tests. (**A**) Au/Ag alloyed nanocages as label-free colorimetric reporters for detection of CEA: (**i**) schematics showing the sensing principle. ALP: alkaline phosphatase; AA-P: l-ascorbic acid 2-phosphate; DHA: l-dehydroascorbic acid; (**ii**) detection results of CEA standards. Adapted with permission from ref [82]. Copyright 2021 American Chemical Society. (**B**) Au nanorods (Au NRs) as label-free colorimetric reporters for detection of HIV antigen: (**i**–**iii**) working principle of the Au NRs-based, microfluidic-integrated multicolor immunosensor for HIV antigen detection. Adapted with permission from ref [83]. Copyright 2020 American Chemical Society.

In a recent work by Xia et al. (see Figure 7A), Au/Ag alloyed nanocages are used as label-free colorimetric reporters for the detection of human carcinoembryonic antigen (CEA, a cancer biomarker) [82]. In this detection system, CEA is specifically captured by antibodies that are labeled with alkaline phosphatase (ALP). ALP can effectively catalyze the formation of ascorbic acid that induces the growth of Ag on the inner surfaces of Au/Ag nanocages. As the amount of Ag inside the nanocages is increased (which is correlated to CEA concentration), a distinct color change from light blue to blue, violet, magenta, and orange, can be visualized. As such, the concentration of CEA in a sample can be conveniently determined by comparing the color of assay solution with the color chart of CEA standards of known concentrations. It should be noted that, compared to the growth of Ag on the surface of solid NPs (e.g., Au nanospheres and nanorods), the growth of Ag inside Au/Ag nanocages is more efficient in tuning the color of NP suspension. This advantage ensures a high detection sensitivity of the Au/Ag nanocages-based detection platform.

In another work by Yang et al. (see Figure 7B), the color change of NP suspension was achieved through chemical etching [83]. Specifically, target antigen HIV-1 p24 was specifically captured by horseradish peroxidase (HRP)-labeled antibodies. HRP-catalyzed oxidation of 3,3′,5,5′-tetramethylbenzidine (TMB) can quantitatively mediate the etching of Au nanorods (Au NRs). The aspect ratio (length/width) of Au NRs was reduced as the extent of etching was increased, which led to various color changes. The assay was performed in a microfluidic platform that enables the integration of all analytical processing within one small chip, making the detection technique particularly suitable for POC testing.

### 3.6. NM NPs-Based POC Tests of Other Mechanisms

In addition to the above mentioned systems, POC tests can be designed and established by taking advantage of other properties of NM NPs through various mechanisms. For example, the average hydrodynamic size of NM NPs can be measured by dynamic light scattering (DLS). The measured size is highly sensitive to the change in the refractive index of surrounding medium of NPs and the coupling or aggregation of NPs [84]. Therefore, NM NPs can be employed for the development of DLS-based POC biosensors. NM NPs are also used in electrochemical biosensors that rely on amperometry or voltammetry techniques [85]. In this approach, NM NPs can enhance electrochemical signal through various mechanisms, such as increasing the loading of electrochemically detectable species and catalyzing the electrolysis of a large amount of substrate [86]. NM NPs of ultra-small sizes (<2 nm), possess fluorescent properties, allowing for the development of fluorescent biosensors [87,88]. In some recent studies, NM NPs were used for developing biosensors with creative mechanisms. For instance, Au nanorods are responsive to the acoustic field, which can induce particle aggregation [89]. Such induced aggregation can be integrated with Raman enhancement for sensitive and rapid biosensing.

## 4. Social Impact

NM NP-based POC testing technology possesses great potential to address disparities in health care. Healthcare disparities, generally considered as the differences in access, utilization, and the quality of care among population groups, affect millions of people in the United States [90]. Underserved populations, such as racial and ethnic minorities, low-income individuals, uninsured or underinsured people, and rural populations, have been disproportionately impacted by healthcare disparities [91]. These populations are found to have worse access to care and/or receive poorer care quality [91,92,93]. Access to care pertains to the ability to obtain the needed and optimal care in a timely manner [94,95]. Research has identified multidimensional barriers to care, including affordability (e.g., high healthcare cost, no or inadequate insurance coverage), availability (e.g., lack of or insufficient facilities, shortage of qualified personnel), and accessibility (e.g., transportation challenges, long travel time, language barriers) [91,95,96,97]. Even among those who initiate healthcare dialogue or treatment, disparities in the quality of care (e.g., receipt of person-centered, coordinated, affordable, safe, and effective care) still exist and continuously pose challenges for the continued treatment engagement and optimal care outcomes [91]. Healthcare disparities can not only lead to adverse health impacts for individuals experiencing disadvantages, but also have a negative financial impact on the entire society due to unnecessary healthcare expenditures (e.g., costs associated with treating severe illnesses, emergency room visits, hospitalization), as well as lost workforce productivity [93,98]. There is clearly a pressing need to reduce healthcare disparities to improve the overall health of the nation’s population.

NM NP-based POC testing can play a critical role in expanding access to quality care. For instance, this testing technology can rapidly and effectively detect various pathogens and biomarkers, while also being less reliant on major equipment and highly trained technicians, as compared to PCR testing [99,100,101]. This is particularly beneficial for individuals living in rural, remote, and/or economically disadvantaged areas where sophisticated testing resources are inadequate or unavailable. Moreover, this type of user-friendly test can be used conveniently at home, which can help eliminate the transportation barriers faced by persons with limited mobility, people with poor access to a vehicle, and those bearing a higher burden of travel for care (e.g., older adults, individuals with disabilities, low-income individuals, racial and ethnic minorities, and residents of rural communities) [99,102,103]. Moreover, this low-cost testing helps address the affordability issue, one prominent barrier to care, for individuals and families, particularly those with no or insufficient insurance coverage [91]. It is estimated that nearly 2% of U.S. people delay obtaining care due to transportation barriers and approximately 30% forgo or delay getting care due to cost [103,104]. Increasing the use of this efficient, convenient, and inexpensive NM NP-based POC testing technology can aid in the early diagnosis, monitoring, and treatment of diseases, lessening individuals’ risk of developing severe symptoms and negative outcomes, and reducing preventable costs within the healthcare system and the society as a whole [90,95].

Furthermore, the COVID-19 pandemic has highlighted the important role and expanded the rise of this rapid, sensitive, user-friendly, and affordable testing technology. Given the fact that COVID-19 is highly contagious and can be spread asymptomatically or pre-symptomatically, having an increased capacity for quick, reliable, and large-scale testing is pivotal in the rapid detection of COVID-19 infections and the timely implementation of infection control measures (e.g., isolation, contact tracing) to prevent community spread and disease outbreak [13,99,105,106]. It can also provide critical information to guide speedy decision-making (e.g., on triage, referral) and appropriate treatment which helps reduce the burden on healthcare systems [106]. As such, future work is recommended to leverage the testing methods, infrastructures, and innovative technologies (e.g., smartphone apps, telehealth) that were rapidly developed during the COVID-19 pandemic to improve community-based public health surveillance and prepare for future infectious diseases.

## 5. Concluding Remarks

In this article, we have discussed recent advancements in noble metal nanoparticles (NM NPs)-based POC testing. Because of their unique features, such as outstanding properties, facile synthesis, and excellent stabilities, NM NPs are particularly suitable for developing nanobiosensors for POC testing. Specifically, NM NPs are utilized as versatile and sensitive transducers to generate various detectable signal through different mechanisms such as catalysis, plasmonics, photothermal effect, and SERS. Notably, the properties and thus signal from NM NPs can be optimized by carefully controlling their physicochemical parameters (e.g., size, shape, internal structure, and elemental composition). The NM NPs-based POC tests are greatly beneficial to society because they provide the public with widespread access to low-cost and effective diagnostics. Significantly, it holds great potential in addressing healthcare disparities and improving the health opportunities and outcomes of many. In addition, it is a valuable tool in the prevention, diagnosis, and monitoring of significant infectious diseases, such as COVID-19.

Despite successful demonstrations and promising progresses, there are still challenges and unmet needs in this field that deserve to be addressed in the future. For example: (*i*) while NM NPs-based POC tests (e.g., LFA) could be simple and easy-to-operate, their sensitivities oftentimes are lower than those sophisticated instrument-based diagnostic techniques. It is challenging yet worthwhile to retain the simplicity of POC testing and meanwhile improve its sensitivity. For instance, the analytical sensitivity of Au NPs-based LFA of COVID antigen was found to be much lower than for reverse transcription-polymerase chain reaction (RT-PCR) tests [107], which may lead to delayed testing; (*ii*) many NM NPs-based POC tests can only provide qualitative or semi-quantitative test results due to the lack of instrument for quantification of detection signal. The returning of a simple “yes or no” answer may not be sufficient for physicians to make medical decisions. In the detection of cancer biomarkers, for instance, quantitative test results are often needed to determine whether the level of certain biomarker in a patient exceeds the cutoff point; (*iii*) researchers are facing challenges in reliably producing high-quality NM NPs. Good batch-to-batch reproducibility is critical to ensure consistent performance of NM NPs-based POC tests. Currently, some synthetic systems for NP production (especially those involving multiple reagents and complicated reaction mechanisms) have significant batch-to-batch variabilities.

The technological revolution and rapid advancement of other related fields reveal new opportunities for the development of advanced NM NPs-based POC tests. Recent efforts toward biosensor miniaturization make POC testing techniques more accessible and/or capable of quantitative analysis. For instance, the uses of handheld devices (e.g., portable Raman spectrometer) and microfluidic platforms have been demonstrated to be effective strategies to develop miniaturized NM NPs-based POC tests. With appropriate setups, personal smartphones can be used for quantitative analysis of POC tests and storage of test results. NM NPs can be coupled with other materials to achieve innovative designs for POC testing. For example, coupling NM NPs with magnetic nanoparticles enables facile separation of target biomarkers from a sample, which eliminates the interferences from complex biological matrices and thus ensures a high signal-to-noise ratio [108]. The knowledge of other disciplines can be used to maximize the capability of NM NPs-based POC tests. For example, machine-learning-based image processing method was used for digital signal analysis in an Ag NP-based plasmonic biosensor [109]. Compared to conventional image processing methods, machine-learning-based image processing is more rapid and accurate, making it suitable for rapid and high-throughput detection. Ultimately, we hope this article can be useful resource to scientists in both academia and industry who are committed to developing advanced POC diagnostic technologies.

## Data Availability

Not applicable.
